# Scope of nanotechnology in ovarian cancer therapeutics

**DOI:** 10.1186/1757-2215-3-19

**Published:** 2010-08-06

**Authors:** Murali M Yallapu, Meena Jaggi, Subhash C Chauhan

**Affiliations:** 1 Cancer Biology Research Center, Sanford Research/USD, Sioux Falls, SD 57104, USA; 2 Department of Obstetrics and Gynecology, Sanford School of Medicine, The University of South Dakota, Sioux Falls, SD 57105, USA; 3 Basic Biomedical Science Division, Sanford School of Medicine, The University of South Dakota, Sioux Falls, SD 57105, USA

## Abstract

This review describes the use of polymer micelle nanotechnology based chemotherapies for ovarian cancer. While various chemotherapeutic agents can be utilized to improve the survival rate of patients with ovarian cancer, their distribution throughout the entire body results in high normal organ toxicity. Polymer micelle nanotechnology aims to improve the therapeutic efficacy of anti-cancer drugs while minimizing the side effects. Herein, different types of polymer micelle technology based nanotherapies such as PLGA, polymerosomes, acid cleavable, thermosensitive, pH sensitive, and cross-linked micelles are introduced and structural differences are explained. Additionally, production methods, stability, sustainability, drug incorporation and drug release profiles of various polymer micelle based nanoformulations are discussed. An important feature of polymer micelle nanotechnology is the small size (10-100 nm) of particles which improves circulation and enables superior accumulation of the therapeutic drugs at the tumor sites. This review provides a comprehensive evaluation of different types of polymer micelles and their implications in ovarian cancer therapeutics.

## Introduction

Ovarian cancer is the fifth most prevalent cancer among women with a life time risk of 1.4 to 1.8% for women living in the US. There are no early symptoms for ovarian cancer which hinders detection until it reaches advanced stages. Survival of the patients is primarily dependent on the disease stage of the patients. For example, stage I, II, III, and IV ovarian cancer have median 5-year survival rates of approximately 93%, 70%, 37%, and 25%, respectively [1,2]. Diagnosed ovarian cancers can be treated by eliminating the cancerous tissue through surgery and care must be taken to prevent the disease from recurring. Surgery alone is effective for only stage I disease, whereas chemotherapy is required in all other stages of ovarian cancer [3]. Therefore, our current review article is focused on the concept of improving the efficacy of ovarian cancer therapeutics using polymer micelle nanotechnology approaches.

### Chemotherapy Agents used for Ovarian Cancer Treatment

**Figure 1:**
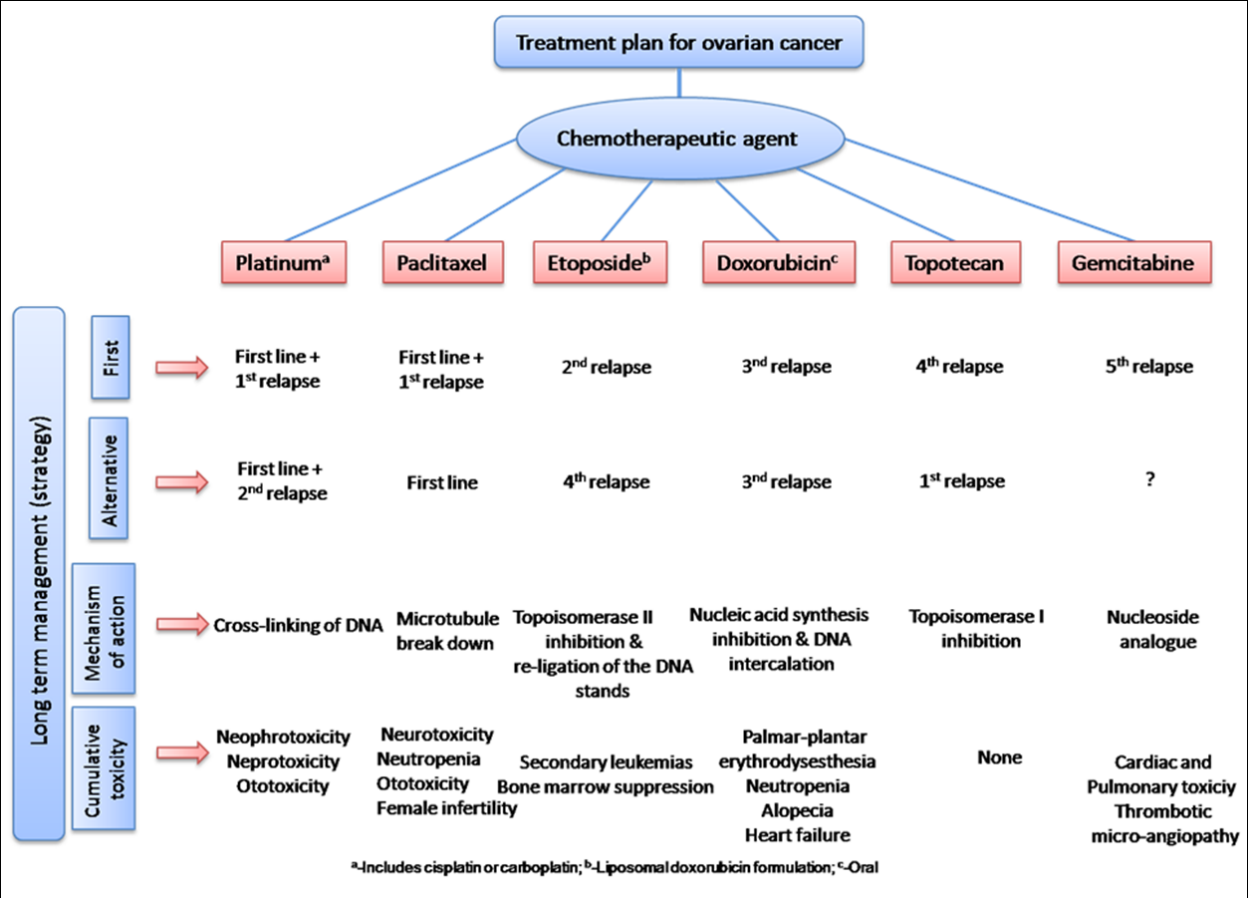
**Long term treatment strategy for ovarian cancer using various anti-cancer drug combinations**.

Chemotherapy helps to improve the overall survival of patients with ovarian cancer. Many chemotherapeutic agents (anti-cancer drugs) are available, including cisplatin (CP), paclitaxel (PTX), doxorubicin (DOX), decitabine (DB), gemcitabine, and their combinations for ovarian cancer treatment. There is significant interest in identifying novel therapeutic agents and improving the efficacy of existing therapeutic modalities. A number of randomized trials treating advanced ovarian cancer using a combination chemotherapy with HEXA-CAF (hexamethyl melamine (HMMA), cyclophosphamide (CPP), methotrexate (MTX) and fluorouracil (FU)) have achieved higher survival rates than using a single therapeutic agent [4]. Other clinical studies using cisplatin, adriamycin, and cyclophosphamide were initiated for stage III and IV ovarian cancer [5-8]. Nevertheless, these trials have not shown a significant benefit of one type of chemotherapy over another. Cisplatin and carboplatin (CBP) have been the most effective chemotherapeutic regimens for more than two decades [9,10]. The majority of current treatment approaches use platinum-containing compounds such as cisplatin, oxaliplatin and transplatin [11-14]. Additionally, paclitaxel (Taxol, TAX) has been recognized as the most efficient chemotherapeutic agent for relapsed ovarian cancer [15]. Doxorubicin, in the form of doxorubicin HCl liposome injection (Doxil or Adiramycin^®^), has also been considered to be an effective therapeutic agent for many years [16]. A combination of doxorubicin, cyclophosphamide, and cisplatin resulted in an increase of 6% in the survival rate of patients versus a treatment of only cyclophosphamide or cisplatin [17]. Gemcitabine has shown positive response in patients who are resistant to cisplatin therapy and it is also a well tolerated drug [18]. An overall 74% response rate was achieved using a combination of gemcitabine with platinum (Gemplat or GemCis) for recurrent carcinoma [19-22]. A flow chart outlining the management of chemotherapeutic strategies is provided in the Figure 1.

It is evident from Figure 1 that, most of the chemotherapeutic drugs have demonstrated a significant therapeutic outcome, at the same time they exhibited adverse side effects [23]. Further, they are not effective in treating the recurrence of ovarian cancer. Importantly, ovarian cancer patients are often initially responsive to these therapeutic modalities but eventually become resistant to therapy. Therefore, drug resistance remains the major obstacle in ovarian cancer treatment. One way to improve the efficacy and specificity of chemotherapeutic agents is through nanotechnology based formulations (encapsulated, conjugated, or entrapped/loaded forms in nanocarriers or drug delivery vehicle/vectors). Nanotechnology mediated therapies promote a controlled delivery of chemotherapeutic drug(s) in a targeted way which directly acts on the cancer site for prolonged periods of time with minimal or no normal organ toxicity. Therefore, this review will focus on the design of nanotechnology formulations for ovarian cancers treatment.

### Nanotechnology in Drug Delivery

**Figure 2:**
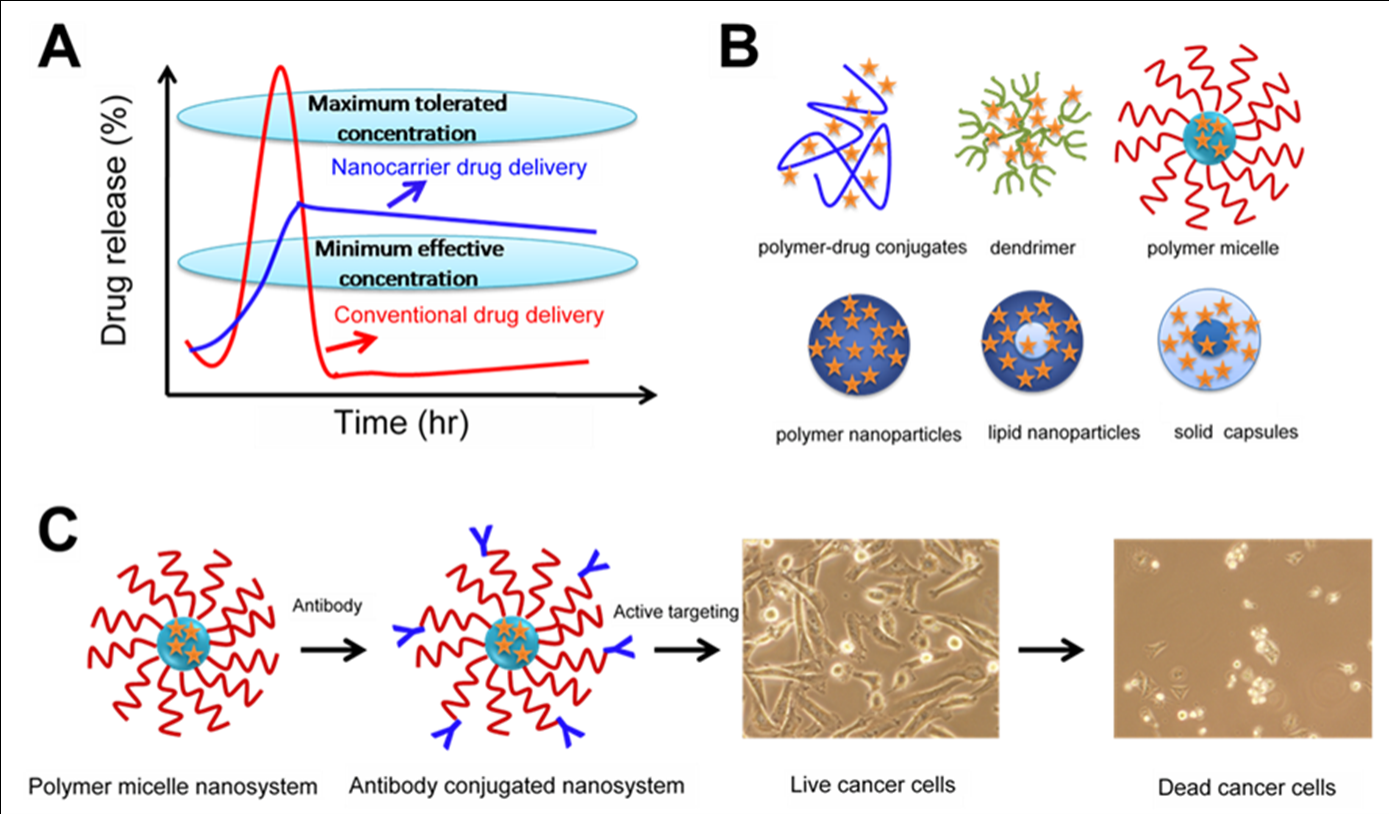
**Improved and sustained therapuetic effect of chemotherapuetic agents using nanotechnology.** (A) Oral or intravenous route delivery of conventional formulations and nanocarriers. (B) Pictorial structures of various drug delivery devices such as polymer-drug conjugates, dendrimer, polymer micelle, polymer nanoparticles, and lipid nanoparticles/capsules. (C) Immunoconjugate nanosystems route for improved therapeutic efficacy.

Chemotherapeutic agents in solution or polymer solution which are delivered orally or intravenously have poor pharmacokinetics with a narrow therapeutic window (Figure 2A). These agents reach a maximum tolerated concentration immediately and then are eliminated from the blood. An ideal drug formulation with maximum benefits for patients should release at a minimum effective concentration over a period of time. Nanotechnology promises to play an important role in satisfying these aspects as a drug delivery carrier/vector (Figure 2B). Nanotechnology based drug carriers such as polymer-drug conjugates, dendrimer, polymer micelles, carbon nanotubes, lipid/solid nanoparticles, and polymer nanoparticles have numerous benefits over conventional methods. Nanotechnology based therapeutics have been proven to improve drug efficacy, reduce toxicity in healthy tissue, and improve patient compliance. Many of these nanoparticles are currently in use for cancer therapies [24]. A list of clinical and preclinical trials of these nanotechnology based formulations have been reviewed by Quan et al. [25]. The design of a universal nanotechnology formulation with chemotherapeutic agents is extremely crucial. A successful formulation, one that acts as a good therapeutic carrier for the cancer therapies, would exhibit the following features: (a) stable in the physiological environment, (b) longer circulation life time (c) avoid opsonization and reticuloendothelial system (RES) process, (d) promote endocytosis, and (e) enhance tumor uptake. The specificity of these formulations can be further enhanced by the conjugation of antibodies to the nanoformulations and these immunoconjugated formulations will have a better therapeutic efficacy over other drug formulations (Figure 2C).

### Drug Delivery Approach

**Figure 3:**
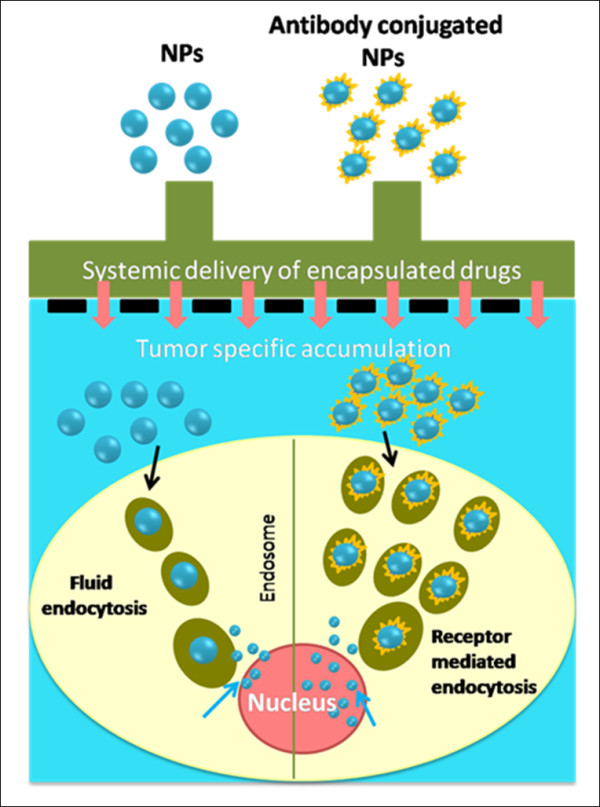
**A schematic representation of the strategy to target cancer cells using nanoparticles and immunoconjugated nanoparticles**. Passive targeting occurs through drug loaded nanoparticles and active targeting is achieved with antibody conjugated nanoparticles.

Chemotherapeutic agent(s) or anti-cancer drug(s) delivery to tumors can be achieved by either a passive or an active mechanism. These mechanisms are shown in a pictorial representation in Figure 3. The passive targeting takes place through the diffusion into tumors or angiogenic tumor vasculatures which have leaky vessels with smaller gaps of 100 - 2000 nm. The nanoformulations (drug loaded nanoparticles) have more interstitial access to the tumor and enhance the retention in tumors. The leaky vasculature promotes the uptake of nanoformulations by the tumors, which become entrapped inside, and due to impaired and poor lymphatic drainage, promotes Enhanced Permeation and Retention (EPR) index. In addition, the size and charge of nanoparticles dictates the passive targeting to the tumors [25-28]. In comparison, the active targeting mode utilizes the conjugation of nanoparticles to immunogens (antibodies or targeting moieties). Delivery of drugs can be improved through tumor specific antibody conjugated nanoparticle system (active targeting) over simple drug loaded nanoparticle system (passive targeting). First, the transport of nanoparticles uptake by the tumor site is increased by longer circulation as a result of the EPR effect. Secondly, the targeting moiety assists in endocytosis of nanoparticles which, in general, increases internalization of nanoparticles for an improved therapeutic effect [29,30]. This targeting approach is promising which has shown enhanced therapeutic effects in animal models *via *substantial increase in nanoparticles internalization in cancer cells [31,32]. In addition to anti-cancer drug delivery, the internalization of nanoparticles is also an important factor in gene, siRNA, DNA and biomacromolecular delivery. Therefore, combination of controlled and targeted delivery improves the efficacy of delivering drugs, genes and biomolecules. In this review we focused on current nanoformulations, especially on polymer micelle nanosystems which have been recognized for their special characteristics.

### Polymer Micelle Nanotechnology

Polymer micelle nanotechnology based delivery of chemotherapeutic agents, imaging agents, biomacromolecules and radionuclides in a tumor-targeted way may enhance diagnosis as well as the outcome of cancer therapy [33]. In this direction, a few clinical trials of various polymer micelle nanotechnology therapies are in the development stage [34]. To describe simply, polymer micelles are formed by a hydrophobic core layered with hydrophilic chains through a spontaneous self-assembly of block or graft copolymers [35,36]. The primary function of polymer micelles is to protect and improve the solubility and stability of hydrophobic (lipophilic) drugs. It has been shown that the aqueous solubility can be increased up to 30,000-fold [37,38]. In our studies, we have demonstrated that β-cyclodextrin, poly(β-cyclodextrin), and polymer nanoparticle curcumin assemblies can improve stability of curcumin by 6-8 fold [39-41]. This higher stability occurs when the drug molecules partition into the hydrophobic core of the polymer micelles and a protective hydrophilic shell interface acts as external medium.

**Figure 4:**
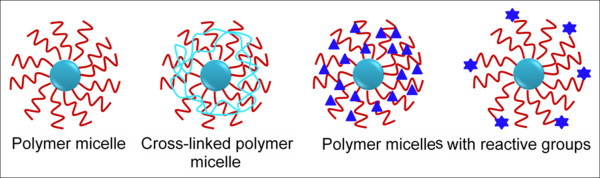
**Different types of polymer micelle formations through the self-assembly process**. This process is always favored by hydrophobic-hydrophobic interactions within the block copolymers. The core is completely hydrophobic which can be used to load anti-cancer drugs. Reactive functional groups can be utilized for antibody conjugations. Illustrations are based on their chemical structures.

There are a number of hydrophobic core-forming biocompatible and biodegradable polymer micelles, such as, poly(ethylene-*co*-propylene-*co*-ethylene oxide) (PEO-*b*-PPO-*b*-PPO) or poly(ethylene-*co*-propylene oxide) (PEO-*b*-PPO), poly(lactic acid) (PLA), poly(_D,L_-lactide) (PDLLA), poly(lactic-*co*-glycolic acid) (PLGA), poly(ε-caprolactone) (PCL), poly(hydroxybutyrate) (PHB), and poly(beta-benzyl L-asparate) [42-47] being used in drug delivery applications. The formation of micelles of these polymers is feasible only at a specific concentration (i.e., critical micelle concentration, CMC). The polymer micelle with a lower CMC value is a better choice for these applications. Figure 4 schematically presents different models of formation of polymer micelles based on their self-assembly mechanisms.

### Conventional Polymer Micelles

**Figure 5:**
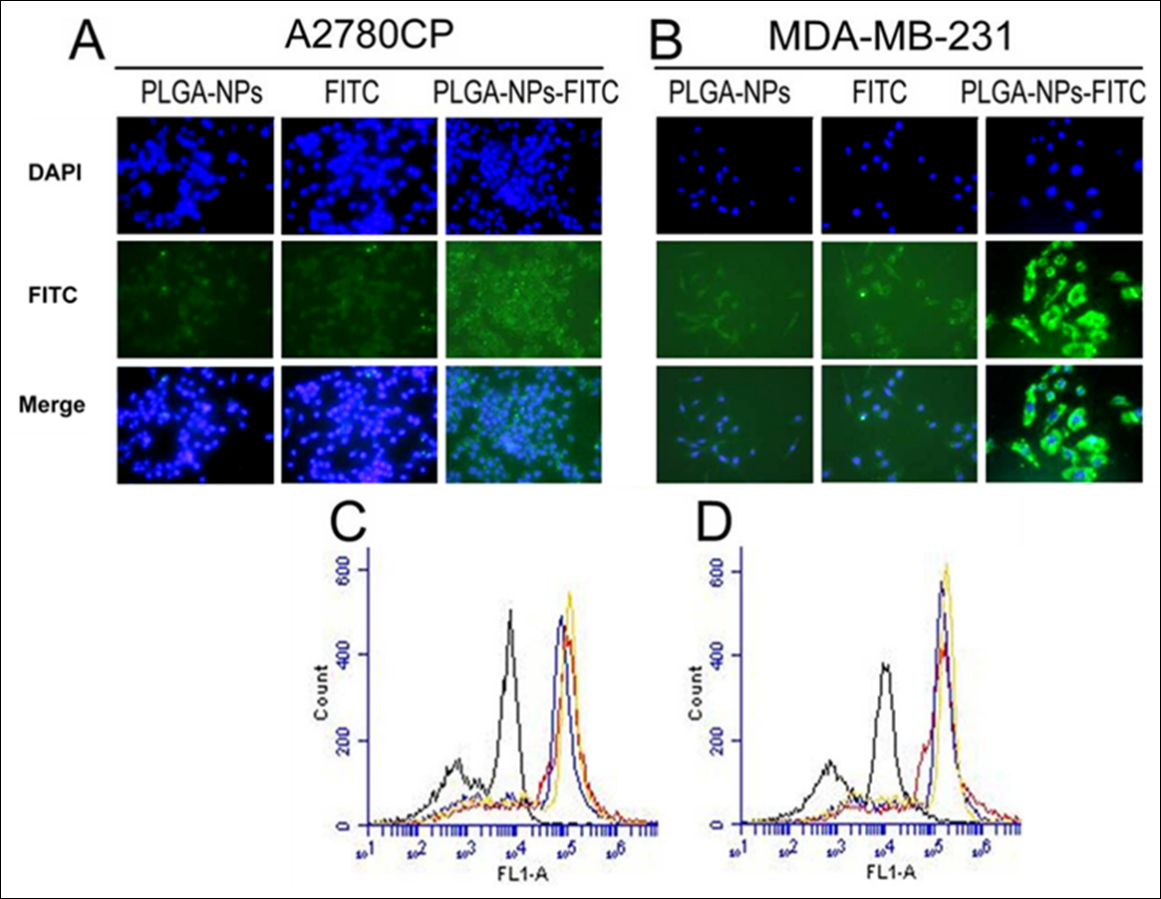
**Uptake of nanoparticles by cancer cells**. (A-B) Fluorescence images of A2780CP and MDA-MB-231 cells treated with PLGA NPs, FITC in solution and FITC loaded PLGA NPs. Nuclei are stained blue with DAPI. (C-D) Fluorescence levels in A2780CP and MDA-MB-231 by Flow Cytometer (Control cells, black line; nanoparticles in cells, blue line; FITC in cell, red line; and FITC-nanoparticles in cells, yellow lines).

A number of natural or synthetic di-block or tri-block copolymers which are biodegradable/biocompatible in nature, have been utilized to load various drugs/biological molecules. Among them, poly(lactic-*co*-glycolic acid) (PLGA) generated micelles are well known. In addition, the parent PLGA polymer is FDA approved for use in industry and medicine. Structurally varied nanoformulations, such as comb-like amphiphilic PLGA-*b*-poly(ethylene glycol) methacrylate (PLGA-*b*-PEGMA) copolymer, PLGA-*b*-poly(ethylene glycol)-*b*-PLGA (PLGA-*b*-PEG-*b*-PLGA) tri-block copolymer, three-arm and four-arm star-shaped PLGA-*b*-PEG block copolymer micelles are available for drug delivery applications [48-50]. In addition, Park et al. [51] recently developed a surface cross-linking PLGA-*b*-PEG copolymer to improve the overall stability of polymer micelles utilizing a shell layer of vinyl pyrrolidone. A natural carbohydrate polymer (i.e. hyaluronic acid (HA) copolymer) can be utilized as target specific micelle carriers for doxorubicin (DOX) by conjugating to PLGA polymer [52]. This formulation allowed loading of 4.8-7.2 wt.% DOX (i.e., DOX-HA-g-PLGA) which exhibited 5.2-fold greater cytotoxicity in the cancer cells over free DOX (IC_50 _value of DOX-HA-*g*-PLGA = 0.67 mg.mL^-1 ^and free DOX = 3.48 mg.mL^-1^). Similarly, a mixed micelle nanoformulation of DOX loaded TPGS/PLGA-*b*-PEG-*b*-FOL (TPGS = α-tocopheryl succinate esterified to polyethylene glycol 1000 and FOL = folate) has shown higher cellular uptake of DOX, which resulted a higher degree of apoptosis in drug-resistant cancer cells. Nanoformulation of PLGA coated with poly(L-lysine)-PEG-folate conjugates has shown an enhanced cellular uptake *via *folate receptor-mediated intracellular delivery [53]. Our data also suggest that PLGA formulations combined with poly(vinyl alcohol) (PVA) achieved intracellular uptake and exhibited improved therapeutic effects of curcumin in cisplatin resistant ovarian (A2780CP) and metastatic breast (MDA-MB-231) cancer cells (Figure 5) [41].

### Other Type of Polymer Micelle Nanoparticles

All drug delivery carrier properties are determined by their stability, solubility, surface charge and type of functional groups which facilitate the encapsulated drug release and targeting characteristics to tumor cells. Pluronic polymers (i.e., poly(ethylene oxide)-*b*-poly(propylene oxide)-*b*-poly(ethylene oxide or PEO-*b*-PPO-*b*-PEO) are known as easily forming micelle drug carriers with a 40 nm diameter. These micelle nanocarriers have the ability to increase the solubility of various hydrophobic anti-cancer drugs as well as enable passive targeting to the solid tumor. Studies have also demonstrated that pluronic micelles promote enhanced cytotoxic activities of various anti-cancer drugs by sensitization of cancer cells attributed to the inhibition of P-glycoprotein (P-gp) activity by depletion of adenosine-5'-triphosphate (ATP) [54]. Poly(ethylene oxide)-linked poly(ethylene imine) (PEO-*l-*PEI) micelle gel is a good example which binds oligonucleotide (ODN) molecules and its delivery is enhanced through receptor-mediated delivery. In general, ODNs are useful therapeutic agents which suffer from severe enzymatic degradation by nucleases. Encapsulation of ODNs in PEG/PEI micelles not only regulated the growth of ovarian cancer cells (A2780) but also lowered ODN concentrations and resulted in significant tumor growth suppression *in vivo *[54,55]. Similarly, stable micelle formulations of 5'-triphosphates of cytarabine (araCTP), gemcitabine (dFdCTP), and floxuridine (FdUTP) in PEG-*l*-PEI networks have been proven to accumulate faster and inhibit tumor growth *in vivo *[56]. Curcumin-casein micelle complexes not only exhibited higher cytotoxicity against HeLa cells but were also capable of damaging cell nucleus as a result of apoptosis at a concentration of 30 μM curcumin [57]. These complexes were also more efficiently internalized in the cells. In our recent investigations [39,40], we have proven that the natural anti-cancer and cancer prevention agent, curcumin, is effective in therapies with self-assembly or nano self-assembly formulations of β-cyclodextrin or poly(β-cyclodextrin).

PEG/PDLLA-Taxol combination (Genexol^®^-PM) is a formulation with high anti-tumor efficacy in human ovarian cancer cell line (OVCAR-3) [58]. In another report, triptolide (TP) loaded PDLLA/PEG nanocarrier was shown to significantly inhibit tumor growth *via *i.v. injections at the dose levels of 0.0375, 0.075 and 0.15 mg/kg, and their inhibition rates were 42.5%, 46.0% and 49.9%, respectively. Hydrolyzable polyesters of PCL and PDLLA are useful formulations to encapsulate paclitaxel, ellipticine, and doxorubicin drugs [59-61]. Further, a novel poly(ethyl ethylene phosphate) (PPE, polyphosphorus ether) and PCL biodegradable triblock copolymer micelles were developed as drug carriers [62]. These micelles are biodegradable, cytocompatible, small sized particles and show improved drug loading efficiency with an increase of PPE molecular weight. The advanced features of these micelles result in more flexibility and their physico-chemical properties can be adjusted through changing the side group conjugation to phosphorus [63]. Another biocompatible micelle (i.e., poly[2-(methacryloyloxy)ethyl phosphorylcholine] or MPC) conjugated with folate targeting moiety to poly[2-(diisopropylamino)ethyl methacrylate] (DPA) (i.e., MPC-DPA-FA), demonstrated a 2.5-fold increase in tamoxifen and paclitaxel uptake [64]. Additionally, cationic polymer micelles can be effectively mediated through endosomal rupture or degradation (i.e., "proton sponge" effect) but often failed at *in vivo *studies due to rapid clearance from the circulation. Therefore, polymerosomes were developed which are cationic polymer micelles that shield the positive charge with a neutral polymer (such as PEG) coating. For example, polyelectrolyte complex (PEC) micelles with luteinizing hormone-releasing hormone (LHRH) peptide exhibited enhanced cellular uptake by increasing VEGF siRNA gene silencing efficiency *via *receptor-mediated endocytosis compared with those without LHRH on LHRH receptor overexpressing ovarian cancer cells (A2780) [65]. Epidermal growth factor (EGF)-conjugated MePEG-*b*-PCL micelles can be delivered at a concentration 13 times more potent than free EGF [66].

### pH Sensitive and Acid Cleavable Polymer Micelle Nanoparticles

The main advantage of these micelles is that encapsulated drugs are burst release in the acidic intracellular compartments such as endosomes or lysosomes. These formulations improved anti-tumor activity through intracellular pH-sensitive drug delivery [67]. Additionally, their folate conjugation was proven to enhance *in vivo *anti-tumor efficacy at lower effective doses [68]. Furthermore, pH-sensitive micelles poly(L-hystidine)-*b*-PEG and PLA-*b*-PEG-*l*-FOL (PHSM-f) were superior compared to free and conventional polymer micelles [69]. The *in vivo *experiments using a sensitive micelle system also demonstrate accumulation of particles at the tumor site and tumor regression was 4-5 fold greater than free DOX after 27 days from the first i.v. injection. One study noted that the half-life of DOX in the pH sensitive micelles increased about 6-fold from free DOX in PBS and plasma media. Their uptake at pH 6.8 was 5 times more than at pH 7.4, indicating that the drug release triggered by the reduced tumor pH was effective after the micelles were accumulated by the EPR effect. Another novel tetra-block copolymer [poly(ethylene glycol)-*b*-poly(L-histidine)-*b*-poly(L-lactic acid)-*b*-poly(ethylene glycol)] is capable of triggering release of DOX at pH 6.8 (i.e., tumor acidic pH) or pH 6.4 (i.e., endosomal pH) compared to normal pH 7.4 [70]. This triggering or burst release effect is dependent upon the molecular weight of the PLA block existing in the tetra polymer which could be a successful therapy for treating solid cancers or delivering cytoplasmic cargo *in vivo*. A new formulation composed of DOX in PDLLA-*b*-PEG-b-poly(L-histidine)-TAT (transactivator of transcription) micelle was able to expose TAT only at a slightly acidic tumor extracellular pH to facilitate the internalization process [71]. These micelles were tested with the xenograft models of human ovarian tumor drug-resistant A2780/AD, human breast tumor drug-sensitive MCF-7 and human lung tumor A549 in a nude mice model, and all tumors considerably regressed in size after three bolus injections at a dose of 10 mg DOX per kg body weight, at three day intervals, while minimum weight loss was observed. The conjugation of drugs to the acid cleavable micelle polymers facilitated prolonged release of drugs [72]. Doxorubicin-conjugated PLLA-mPEG micelles were more potent because they were taken up within cells with simultaneous rapid release of cleaved doxorubicin into the cytoplasm from acidic endosomes [72]. A Triblock copolymer conjugated with DOX through the end OH groups of copolymers, indicated that hydrazone linkage was cleaved under acidic conditions [73]. This behavior was confirmed by flow cytometry and confocal microscopy which demonstrated the extent of cellular uptake of micelle conjugated DOX and distribution in the cytoplasm, endosomal/liposomal vesicles, and nucleus, while the free drug was localized within the nucleus.

### Cross-linked Polymer Micelle Nanoparticles

Various polymer micelle nanoparticles can control the triggered release of the active therapeutic agents, but most of these polymer micelle nanoparticles have drawbacks as delivery carriers. For example, paclitaxel was readily disassociated from the micelle nanoparticle just after injection into the blood stream [74]. This dissociation may be due to the decomposition of micelles α- and β-globulins and translocation of paclitaxel to the abundant lipid components and carriers in blood [75]. To address this drawback of polymer micelle nanoparticles, a possible strategy is to design cross-linked biodegradable micelles [76]. These micelles can shield drug molecules tightly by the cross-linked corona and biodegradable cross-linking releases the drug from micelles in a controlled manner. Core micelle cross-linked with divalent metal cations display high stability but also exhibit pH-dependant swelling/collapse behavior [77]. These systems have remarkably high platinum loading efficiency (i.e., ~22% wt./wt.) and exhibited slow release of platinum in a sustained manner from the cisplatin-loaded cross-linked micelles in physiological saline. A new formulation based on α-methoxypoly(ethylene oxide)-*b*-poly[*N*-(3-aminopropyl)methacrylamide]-*b*-poly[2-(diisopropylamino)ethyl methacrylate] (mPEO-PAPMA-PDPAEMA), poly(*N*-isopropylacrylamide), and *N*-hydroxysuccinimidyl esters (NHS) prevented the dissolution of micelles due to dilution effects and enabled pH sensitive and potentially cleavable sites for micelle disassembly [78].

### Novel Polymer Micelle Nanotechnology Strategies

Double-hydrophilic block copolymer based micelles have more external hydrophilic behavior which mimics biological fluid, unlike core-shell block copolymers [79,80]. The first hydrophilic charged block copolymer binds to the chemotherapeutic agent and the second hydrophilic block allows for steric stabilization. Polyamino acid-*b*-polyethylene oxide copolymers are excellent examples which can bind the oppositely charged species such as drugs (cisplatin and doxorubicin), proteins or peptides, nucleic acids, and lysozymes [81-84]. Ultrasound sensitive polymer micelle nanoparticles are another modality of drug targeting to tumors by a localized release. These micelles are degraded when subjected to ultrasound into unimers which enhances the cell membrane perturbation [85]. Even a short exposure of 15 to 30 seconds to high-frequency ultrasound leads to a significant increase in the intracellular DOX uptake from pluronic micelles [86].

**Figure 6:**
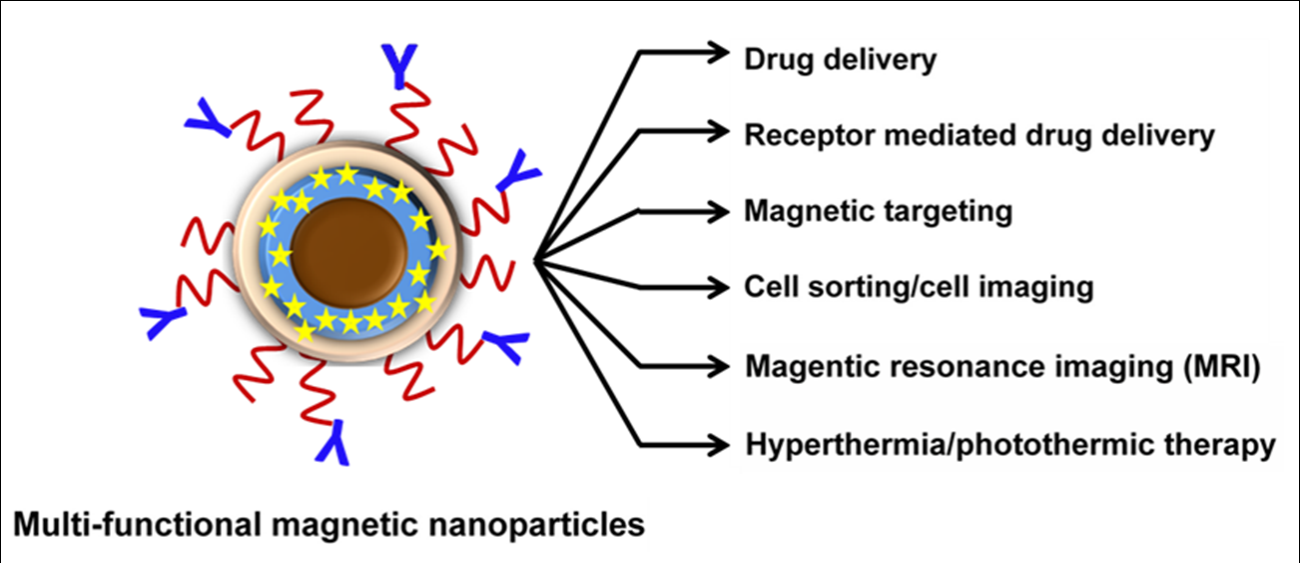
**Multi-functional magnetic nanoformulation with curcumin/photo activator loaded, double layer, antibody conjugation for various medical applications**.

Magnetic nanoparticle based micelles act as drug carriers as well as external magnetic field guides in cancer therapy treatments [87]. Developing micelle-magnetic nanoparticles is a promising alternative. Recently, such formulations were developed to gain different biological functions while using only one formulation [88]. These formulations can be applied not only for drug delivery techniques but also magnetic resonance imaging (MRI), visible targeting, magnetically targeted photodynamic therapy, targeted thermo-sensitive chemotherapy, and luminescence/near-infrared/multi-model imaging applications [89-96]. In this regard, one novel formulation composed of an iron oxide nano-core stabilized with a multi-layer coating could achieve better feasibility in drug delivery, imaging and hyperthermia properties. However, the higher hydrodynamic diameter (> 200 nm) in aqueous medium limits its use in cancer therapeutic applications [97]. Therefore, we have been developing a novel formulation of magnetic nanoparticles composed of iron oxide core that is subsequently coated with β-cyclodextrin (CD) and pluronic F127 polymer (F-127) which possesses anti-cancer drug loading and antibody conjugation features and can be utilized for multi-functional applications (Figure 6). The advantages of this formulation include smaller particle size, relatively lower protein binding, higher drug loading efficiency and enhanced particles uptake in cancer cells without hampering inherent magnetization characteristics.

## Conclusions

Polymer micelle nanotechnology has demonstrated that nanoparticles are capable of loading anti-cancer drugs which can be specifically targeted to tumors through the conjugation of tumor specific antibody/moiety. Multi-functional polymer micelles, including nanogels/magnetic based micelles, possess characteristics which could improve ovarian cancer therapy. These formulations have capabilities of MRI visible targeting, targeted photodynamic therapy, thermosensitive therapy and luminescence/near-infrared/multi-model imaging properties, which will allow tracking and monitoring of nanoformulations and accumulated drug(s) at the tumor site during the therapy procedure.

## Competing interests

The authors declare that they have no competing interests.

## Authors' contributions

MMY drafted the manuscript. MJ and SCC participated in revising the manuscript. All authors have read and approved the final manuscript.
